# Smoking cessation – better together: A retrospective cohort study

**DOI:** 10.18332/tid/162367

**Published:** 2023-05-19

**Authors:** Limor Adler, Shafeek Abu Arar, Ilan Yehoshua, Bar Cohen, Sharon Hermoni Alon, Arnon Shahar, Galia Zacay, Miri Mizrahi Reuveni

**Affiliations:** 1 Health Division, Maccabi Healthcare Services, Tel Aviv, Israel; 2 Department of Family Medicine, Sackler Faculty of Medicine, Tel Aviv University, Tel Aviv, Israel; 3 Department of Family Medicine, Ben-Gurion University of the Negev, Beer Sheva, Israel; 4 Department of Health Science, Ben-Gurion University of the Negev, Beer Sheva, Israel

**Keywords:** smoking cessation, partner habits, health promotion

## Abstract

**INTRODUCTION:**

Smoking is the leading preventable cause of death and illness globally. There is conflicting evidence regarding the association between quitting rates and partners’ smoking status. It is thought that spouses influence one another’s health habits, including smoking. This study aims to evaluate this association in patients who made a smoking cessation attempt with pharmacotherapy.

**METHODS:**

For this Israeli nationwide retrospective cohort study, we randomly selected patients who filled a prescription for varenicline as part of their smoking cessation process and were partnered. The participants were asked to complete a questionnaire 26–52 weeks after the first varenicline purchase. The independent variables were the partner’s smoking status at the beginning of the smoking cessation process and while answering the questionnaire. The outcome was a success in the quitting process.

**RESULTS:**

In all, 226 (50%) participants had partners who smoked at the beginning of the quitting process, and 230 (50%) had non-smoking partners; 178 (39%) participants reported successful smoking cessation. There was a significant difference in success rates depending on partners’ smoking status at the end of the process, with success rates of 39% with a non-smoking partner, 76% with a partner who also stopped smoking, and 31% with a partner who continued smoking (p<0.001). Multivariate analysis showed that having a partner who stopped smoking during the quitting process was associated with higher odds of quitting compared with having a non-smoking partner (OR=4.73; 95% CI: 1.86–12.05).

**CONCLUSIONS:**

This study showed that both partners quitting was associated with increased odds of successful quitting. Health providers should make efforts to engage both partners in smoking cessation.

## INTRODUCTION

According to the World Health Organization, smoking is the leading preventable cause of death and illness globally, with 7 million deaths directly from smoking and 1.2 million deaths resulting from passive exposure to smoke yearly^1^. Medical establishments and professionals worldwide regularly make efforts to encourage smoking cessation and decrease smoking rates. Quitting smoking is the most significant change smokers can make to improve their health outcomes^1,2^.

### Smoking cessation

Among the most common smoking cessation techniques are behavioral therapy (workshops, telephone assistance, website programs, counseling by one’s physician, etc.) and pharmaceutical therapy (nicotine replacement, varenicline, bupropion, etc.)^3,4^. A combination of educational and behavioral treatment with pharmacotherapy is the method of choice for many and has been proven the most effective^3^. Varenicline, an α4β2 nicotine acetylcholine receptor partial agonist that requires a prescription and guidance from a nurse or a physician, is considered effective in supporting the quitting process^3^.

It is thought that spouses influence one another’s health habits, including smoking, alcohol consumption, exercising, cholesterol screening, and more^5^. Several studies have examined the association between spousal smoking status and success in quitting. Falba and Sindelar^5^ reported that when a participant’s partner had quit smoking, the participant was 7.5 times more likely to quit successfully if male, and 8.5 times more likely if female^5^. Additionally, Cobb et al.^6^ found that men and women who were married to a current smoker were less likely to quit smoking, and women who were married to a former smoker were more likely to quit^6^. Foulstone et al.^7^ reported similar results and suggested that partners’ smoking status and relationship satisfaction are essential factors in smoking cessation. However, not all studies show similar results; Palali and Van Ours^8^, for example, found no evidence of a spousal effect on the decision to quit smoking.

This study aimed to evaluate the correlation between quitting rates of individuals who made a smoking cessation attempt using pharmacotherapy and their partner’s smoking habits before and during the cessation process.

## METHODS

### Study design and setting

In this retrospective cohort study, we selected participants from Maccabi Healthcare Services (MHS), Israel’s second-largest health maintenance organization, who had filled a prescription for varenicline between October 2019 and September 2020. We approached participants 26 to 52 weeks after the initiation of treatment and asked them to complete a self-report questionnaire using emails, text messages, and phone calls. The targeted participants attended group counseling (eight 90-minute sessions) or phone counseling (six 30-minute sessions) or received counseling from their general practitioners (GPs)^9^. All these sessions were subsidized and offered to all MHS members. These three forms of intervention will be addressed as the quitting process. The beginning of the quitting process is taken to be the time of the first purchase of varenicline.

### Participants

Participants were randomly selected from all patients who filled a prescription for varenicline 26 to 52 weeks before the commencement of the study, using a simple random method. The inclusion criteria included being an adult (aged >18 years) and an MHS member who purchased varenicline. Prospective participants were sent emails or text messages inviting them to participate and answer an online questionnaire. The questionnaire was not validated but did go through a face validity checkup by primary care physicians that perform smoking cessation counseling. Potential participants who failed to interact with the sent messages were contacted by telephone by MHS and completed the questionnaire as an interview upon providing consent.

### Variables

The questionnaire was created by the authors of this study and included multiple scales and items designed to evaluate participant satisfaction and the efficacy and characteristics of successful and unsuccessful smoking cessation processes in the target population. The rankings included sociodemographic characteristics, medical data, and information about quitting. The respondents were asked about their partner’s smoking status before the quitting process commenced and at the time of answering the questionnaire.

The dependent variable was success in quitting, measured as a binary variable, smoking status at weeks 26 to 52. The independent variable, partner smoking status, was divided into three groups: 1) ‘non-smoking partner’, i.e. a partner who did not smoke at the beginning of the quitting process or at the time the questionnaire was completed; 2) ‘partner who stopped smoking during the quitting process’, i.e. a partner who smoked at the beginning of the quitting process but did not smoke at the time the questionnaire was completed; and 3) ‘smoking partner’, i.e. a partner who smoked at the beginning of the process and at the time the questionnaire was completed or a partner who did not smoke at the beginning of the quitting process but did smoke at the time the questionnaire was completed (we had to combine the different types of ‘smoking partners’ into one exposure group, as there were not enough participants to separate them into two groups).

The independent sociodemographic variables were age, gender, and socioeconomic status (SES). SES is measured on a scale 1–10 (1=lowest to 10= highest) and determined according to residency address using classification done by the Israel Central Bureau of Statistics; we clustered it into three groups: low (1–3), intermediate (4–7), and high (8–10). Medical independent variables included comorbid conditions listed in the medical records: diabetes mellitus, ischemic heart disease (IHD), hypertension, COPD, chronic kidney disease, and oncologic disease.

Independent variables related to the cessation process included quitting method (three options for categorical variable coding: group counseling, phone counseling, and GP counseling), daily consumption of cigarettes before quitting, number of years of smoking, and duration of varenicline therapy (in months).

### Sample size calculation

We assumed a difference of 15% between the groups; a successful quit rate of 35% in the partner who smoked group and 50% in the partner who did not smoke group. To prove a significant difference with a significance level of 5% and power of 80%, a sample of 366 participants was needed (183 in each group).

### Statistical analysis

First, descriptive statistics for both the test and control groups were produced. Later, a series of bivariate analyses, including chi-squared for categorical variables and Mann Whitney for non-normally distributed continuous variables, were conducted to establish which statistically significant correlations existed. Each significantly correlated variable found in the bivariate phase was later checked in a logistic regression model to produce a final model. We fitted the regression model in two blocks: the first with age, gender, and SES group using the ENTER method, while the second block, which was entered using the FORWARD method, included quitting method, comorbidities (diabetes mellitus, hypertension, IHD, COPD, CKD, oncologic condition), number of cigarettes per day, years of smoking, duration of varenicline therapy, partner smoking status, and correlation interaction between gender and smoking status of the partner. Two-tailed analyses were done for all tests, with a probability level <0.05 considered significant. We used Statistical Package for Social Sciences (SPSS) software version 28 for data analysis.

## RESULTS

### Study population

The original cohort comprised 604 participants (604/870, representing a 69% response rate). For this analysis regarding the association between smoking cessation and partners’ smoking habits, a specific sample was extracted to include only relevant participants (i.e. participants partnered at the time of the questionnaire). This final sample consisted of 456 participants: a control group (n=230) of participants with a non-smoking partner and a test group (n=226) of participants with a partner who smoked at the beginning of the quitting process. The test and control groups were similar in most respects (Table 1). Their average age was 45–46 years, and two-thirds of both groups belonged to the intermediate SES group and had similar prevalences of chronic diseases. The number of cigarettes the participant smoked per day (before quitting) and years of smoking were similar in the two groups (approximately 20 cigarettes per day for 23–25 years). However, more males had non-smoking partners, and diabetes mellitus was higher in participants with non-smoking partners.

**Table 1:** Characteristics of patients whose partners smoked or did not smoke at the beginning of the quitting process. A retrospective Israeli study which was conducted in a community setting (N=456)

*Characteristics*	*Partner smoked (N=226) n (%)*	*Partner did not smoke (N=230) n (%)*	*p*
**Sociodemographic**			
**Gender** – male	120 (53.1)	151 (67.5)	0.008
**Age** (years), mean ± SD	45.3 ± 11.2	46.7 ± 12.0	0.208
**SES group**			0.577
Low (1–3)	16 (7.1)	18 (7.8)	
Intermediate (4–7)	156 (69)	148 (64.3)	
High (8–10)	54 (23.9)	64 (27.8)	
**Medical**			
Diabetes mellitus	17 (8.3)	31 (14.8)	0.045
Hypertension	39 (18.9)	48 (23)	0.336
IHD	25 (11.1)	23 (10)	0.939
Missing data	20 (8.8)	21 (9.1)	
COPD	7 (3.1)	8 (3.5)	>0.999
Missing data	20 (8.8)	21 (9.1)	
Chronic kidney disease	14 (6.8)	14 (6.7)	>0.999
Oncologic disease	8 (3.9)	12 (5.7)	0.493
**Smoking cessation**			
**Quitting method**			
Intensive counseling (group)	88 (38.9)	69 (30.0)	0.073
Intensive counseling (telephone)	30 (13.3)	44 (19.1)	
GP counseling	108 (47.8)	117 (50.9)	
**Number of cigarettes per day** (before the quitting process), mean ± SD	20.66 ± 9.1	20.03 ± 10.3	0.488
**Years of smoking**, mean ± SD	23.54 ± 10.8	25.17 ± 12.0	0.128
**Duration of varenicline therapy** (months), mean ± SD	2.18 ± 1.4	2.33 ± 1.4	0.257

*Patients who did not report the smoking status of their partners at the beginning of the quitting process were excluded from this analysis.

### Outcomes

**Figure 1:**
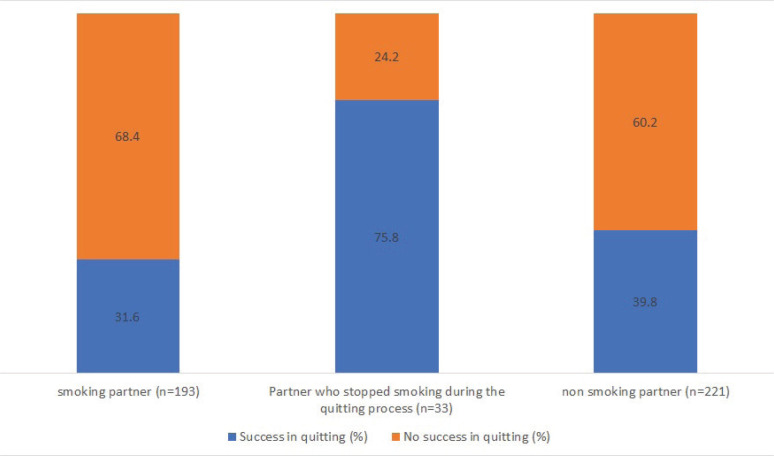
Success in the quitting process, based on the partner’s smoking status

In all, 178 participants reported that they quit smoking (Table 2). The overall rate of successful smoking cessation was 39%. A comparison of quitting rates in association with the smoking status of the partner revealed success rates of 39.8% in the ‘non-smoking partner’ group, 75.8% in the ‘partner who stopped smoking during the quitting process’ group, and 31.6% in the ‘smoking partner’ group (p<0.001) (Figure 1). Several other independent variables, including SES, IHD, the mean number of cigarettes per day (before the quitting process), years of smoking, and duration of varenicline therapy, emerged as significantly correlated with a successful result in the quitting process (Table 2). Participants from the high SES subgroup displayed higher success rates than their counterparts from the other groups (p<0.001). Among the medical variables, only IHD proved significant; participants with IHD were less successful in quitting (p=0.013). The number of cigarettes consumed before quitting, years of smoking, and duration of varenicline therapy were shown to correlate with success in the quitting process (p=0.022, p=0.013, and p=0.031, respectively); smoking fewer cigarettes per day and smoking for fewer years were associated with higher success rates in the quitting process. Duration of varenicline therapy was positively correlated with successful smoking cessation (p<0.001).

**Table 2:** Bivariate comparison of success in quitting process

*Variables*	*Success in quitting (N=178) n (%)*	*No success in quitting (N=278) n (%)*	*p*
Partner*			
**Partner smoking status**			
Non-smoking partner	88 (50.6)	133 (48.7)	
Partner who stopped smoking during the quitting process	25 (14.4)	8 (2.9)	
Smoking partner	61 (35.1)	132 (48.4)	<0.001
**Sociodemographic**			
**Gender** - male	101 (56.7)	170 (61.2)	0.379
**Age** (years), mean ± SD	44.8 ± 11.1	46.7 ± 11.9	0.104
**SES group**			
Low (1–3)	12 (6.7)	22 (7.9)	
Intermediate (4–7)	102 (57.3)	202 (72.7)	<0.001
High (8–10)	64 (36)	54 (19.4)	
**Medical**			
Diabetes mellitus	14 (8.9)	34 (13.2)	0.207
Hypertension	26 (16.5)	61 (23.7)	0.083
IHD	10 (5.6)	38 (13.7)	0.013
Missing data	20 (11.2)	21 (7.6)	0.184
COPD	8 (4.5)	7 (2.5)	0.162
Missing data	20 (11.2)	21 (7.6)	0.490
Chronic kidney disease	7 (4.4)	21 (8.2)	
Oncologic disease	6 (3.8)	14 (5.4)	
**Smoking cessation**			
**Quitting method**			
Intensive counseling (group)	61 (34.3)	96 (34.5)	0.251
Intensive counseling (telephone)	35 (19.7)	39 (14)	
GP counseling	82 (46.1)	143 (51.4)	
**Number of cigarettes per day** (before the quitting process), mean ± SD	19.0 ± 9.5	21.2 ± 9.8	0.022
**Years of smoking,** mean ± SD	22.5 ± 11.7	25.5 ± 11.7	0.013
**Duration of varenicline therapy** (months), mean ± SD	2.5 ± 1.5	2.1 ± 1.2	0.031

Multivariable analysis of the listed variables and their effects on successful smoking cessation yielded several significant results (variables selection methodology and analysis results are available in Table 3). Having a partner who stopped smoking during the quitting process was associated with higher odds of quitting compared with having a non-smoking partner (OR=4.73; 95% CI: 1.86–12.05; p=0.001), while there was a trend towards lower odds of quitting with a smoking partner (OR=0.67; 95% CI: 0.43–1.05, p=0.081). Being in the high SES group and having a relatively longer duration of varenicline therapy increased the chances of successful quitting (OR=2.48; 95% CI: 1.51–4.09; p<0.001 and OR=1.27; 95% CI: 1.08–1.48; p=0.003, respectively). A more significant number of years of smoking was a risk factor for unsuccessful quitting (OR=0.97; 95% CI: 0.94–0.99; p=0.03).

**Table 3:** Multivariable analysis of success in quitting process

*Variable*	*OR (95% CI)*	*p*
**Partner smoking status**		
Non-smoking partner (Ref.)	1	
Partner who stopped smoking during the quitting process	4.73 (1.86–12.05)	0.001
Smoking partner	0.67 (0.43–1.05)	0.081
**Age** (years)	1.01 (0.98–1.04)	0.711
**Gender** (female)	1.19 (0.77–1.85)	0.428
**SES group**		
Low (1–3)	1.44 (0.64–3.24)	0.379
Intermediate (4–7) (Ref.)	1	
High (8–10)	2.48 (1.51–4.09)	<0.001
**Years of smoking**	0.97 (0.94–0.99)	0.03
**Duration of varenicline consumption**	1.27 (1.08–1.48)	0.003

Age, gender, and SES group were entered using the ENTER method. Quitting method, comorbidities (diabetes mellitus, hypertension, IHD, COPD, CKD, oncologic condition), number of cigarettes per day, years of smoking, duration of varenicline therapy, partner smoking status, and interaction between gender/age/duration of smoking/SES and smoking status of the partner were entered using the FORWARD method.

## DISCUSSION

### Main findings

According to the analysis results, both partners quitting was associated with a more than 4.5-fold increase in the odds of successful quitting when using pharmacotherapy (compared to having a non-smoker partner). Additional factors that influenced the success of the quitting process were the duration of varenicline therapy and high socioeconomic status, which were associated with higher success rates in attempting to quit smoking, and a more prolonged period of smoking, which was associated with lower rates of successful smoking cessation.

### Comparison with other studies

The overall quitting rate in our study was 39% after 26 to 52 weeks. This rate is in line with those found in other studies that explored short-term quitting rates with pharmacotherapy^10^.

We found that partners’ smoking habits significantly influenced the success rates in quitting smoking. Having a partner who stopped smoking increased the odds of successfully quitting by >450%. This finding suggests that a shared attempt to quit smoking may be a decisive factor in successful smoking cessation. Falba and Sindelar^5^ offer several explanations for this finding. First, partners tend to have similar health behaviors when they marry, and one partner’s decision to change may influence the other partner. The fact that partners tend to have similar education levels and that education is fundamental in quitting smoking may also explain why shared attempts to quit smoking are often successful. Second, personal and environmental changes that may affect the decision to change a health behavior may be experienced by both partners and influence them (e.g. a change in location, the death of a friend, retirement, etc.). Another possible explanation, suggested by Ruge et al.^11^, is that the partner’s smoking status is associated with the intention to quit smoking. Another mechanism of influence was proposed by Britton et al.^12^, who suggest that the perceived responsiveness of the partner (the perception that the partner understands, validates, and supports the person attempting to quit smoking) has a greater influence than mere support.

In a recent study conducted by Buitenhuis et al.^13^ on single-smoker couples, the involvement of a non-smoking partner in the smoking cessation process did not increase quit rates (33% in mutual attempts vs 30% in individual attempts). In contrast, Luscher et al.^14^ found a link between partners’ smoking status and success in quitting in dual-smoker couples. Emotional and instrumental support are both key to this connection. These two studies suggest that the partner’s influence is more complex than we have understood until now.

In the context of partner smoking status, some studies suggest that gender is a significant factor in the influence of one’s partner, while others found no such connection^7,15–17^. In our study, gender was not significantly associated with success, nor was the interaction between gender and partner smoking status. Generally, women seem to have more difficulty than men in maintaining long-term smoking cessation^16^. Smith et al.^18^ found no differences between men and women regarding desire, plans, or attempts to quit smoking. However, women had 30% lower odds of succeeding in quitting. When medications were used, no such difference was found. Another possible explanation is suggested by Dieleman et al.^19^, whose qualitative study showed that the main barriers to smoking cessation in women were psychological and included emotion and stress. In contrast, for men, the barriers were environmental. It may be more difficult for women than men to overcome these difficulties in the long run.

While most participants in both groups belonged to the intermediate SES group, rates of successful smoking cessation processes were disproportionately increased in the high SES group. Notably, all the offered treatments are subsidized by the state and MHS, meaning that this is no indication of reduced direct access to such services. In a review by Hiscock et al.^20^, participants with low socioeconomic status tend to smoke more, and their attempts to quit are less likely to be successful. They suggest several explanations for this phenomenon, including reduced social support, low motivation to quit, stronger addiction to tobacco, and a lower likelihood of completing courses of pharmacotherapy or behavioral support. Cambron et al.^21^ suggest another explanation, i.e. that participants from lower SES backgrounds are more exposed to pro-smoking social contexts that increase the risk of unsuccessful quitting attempts.

Another noteworthy finding is that while many participants have a comorbidity related to or associated with smoking, these have mainly shown little effect on quitting. In the bivariate analysis, IHD was more prevalent among persons who failed to quit. Yet, in the multivariable analysis, IHD was not included in the final model as a significant variable. A review and meta-analysis conducted by Lovatt et al.^22^ found that most smokers with acute coronary syndrome continue to smoke after the cardiac event.

The compound difficulties of long-term smoking habits are also manifested in the correlation between years of smoking and the results of the quitting process, i.e. the longer one smokes, the lower one’s chances of successfully quitting. While this phenomenon is consistent with existing knowledge regarding the age of the acquisition of smoking habits, one may consider that this effect is multifactorial in its own right and includes aspects of social environment, behavioral habits, and physical dependence^23^.

An additional finding is the effect of varenicline therapy. A longer duration of treatment increases the odds of successful quitting. This finding highlights the importance of adherence to therapy in supporting participants who seek to quit smoking^24^. The method and intensity of the therapeutic framework chosen (intensive group counseling, personal phone counseling, or brief GP counseling) proved insignificant in this study, suggesting it may be best to tailor counseling to individual participants rather than adopt a ‘one-size-fits-all’ policy^9^.

### Strengths and limitations

This study has several strengths, including the relatively large sample size (456 participants), the fact that participants were drawn from across the country, and the high response rate (69%). Another strength of the study is the availability of data regarding participants’ comorbidities and SES from the electronic medical record, which reduced recall and report bias.

This study has several limitations. First, this sample is local, and any conclusions may only be relevant to some countries. Although the sample and response rate are adequate, a larger sample may strengthen the results. Additionally, recruitment for this study was conducted via text messages or phone calls. While an attempt was made to contact potential participants and include as many as possible, a specific subpopulation may have been under-represented, resulting in selection bias. In addition, we did not have information about prior smoking attempts of the participants and whether participants lived with their partners. Dependence on self-reporting as a measure of smoking status may have resulted in reporting bias. Nonetheless, self-reported smoking status is considered reliable^25,26^.

## CONCLUSIONS

We found that the current smoking status of the partner of an individual attempting to quit smoking using pharmacotherapy is associated with a successful quitting process. Smoking status before the quitting attempt was not associated with success, highlighting the importance of awareness of the smoking status of partners in quitting attempts and the impact of shared change. We suggest that GPs and nurses who engage in smoking cessation counseling include this parameter in their routine follow-up of these participants. Other factors that emerged as significant for successful quitting included high SES status and longer duration of varenicline therapy. Additionally, IHD and longer smoking duration were revealed as factors that may negatively affect the success of the cessation process.

## Data Availability

The data supporting this research are available from the authors on reasonable request.
